# Inpatient hospital course and self-reported symptomatology in underweight adults with ARFID compared to age- and sex-matched controls with anorexia nervosa

**DOI:** 10.1186/s40337-023-00912-x

**Published:** 2023-11-20

**Authors:** Irina A. Vanzhula, Erin Wang, Mary K. Martinelli, Colleen Schreyer, Angela S. Guarda

**Affiliations:** Johns Hopkins University School of Medicine, Meyer 101, 600 N. Wolfe Street, Baltimore, MD 21287 USA

**Keywords:** Anorexia nervosa, ARFID, Inpatient treatment, Weight restoration

## Abstract

**Objective:**

Avoidant restrictive food intake disorder (ARFID) has similar prevalence to anorexia nervosa (AN) in adults, but research in this population is lacking. Although inpatient or residential treatment involving nutritional rehabilitation is increasingly recommended for malnourished individuals with ARFID, best practices remain poorly defined. Existing studies on self-reported symptomatology and treatment course and outcome are primarily in child and adolescent cohorts and demonstrate inconsistent findings. This study aimed to compare hospital course and self-reported symptomatology of underweight adult inpatients with ARFID and sex- and age-matched patients with AN.

**Method:**

Underweight adult patients with ARFID or AN admitted to a specialized, hospital-based behavioral treatment program completed measures of body dissatisfaction, drive for thinness, bulimic symptoms, anxiety, depression, and personality traits. Demographic and treatment course data were abstracted from electronic medical records. Patients with ARFID (*n* = 69) were matched to those with AN (*n* = 69) based on sex and age.

**Results:**

Adults with ARFID were closer to target weight at admission, but gained weight at a slower rate, were discharged at lower BMI, and were less likely to reach target weight by discharge than adults with AN. Patients with ARFID reported less weight and shape-related eating disorder, state anxiety, and depression symptoms and lower neuroticism.

**Discussion:**

Adults with ARFID progress through treatment more slowly and achieve less favorable weight outcomes by hospital discharge than patients with AN, but long-term outcomes are unclear. Describing clinical presentations and course of illness of adult ARFID may help inform treatment protocols.

## Abstract (plain language summary)

This study examined hospital course and symptomatology in underweight adults with avoidant/restrictive food intake disorder (ARFID) compared to adults with anorexia nervosa (AN). Both groups were admitted at similar BMI, however compared to patients with AN, those with ARFID gained weight slower and were half as likely to reach target weight by discharge. Adults with ARFID were equally likely, however, to be rated as clinically improved at discharge. Patients with ARFID also reported less symptoms of anxiety, depression or neuroticism than did those with AN, and less weight and shape-related eating disorder symptoms at admission. The reason for slower weight restoration in adults with ARFID may reflect the need for more individualized protocols adapted to meet the unique, often heterogeneous needs of these individuals. Longer-term post-discharge outcomes for adults with ARFID remain unclear and require investigation.

## Introduction

Avoidant/restrictive food intake disorder (ARFID) is defined as a persistent disturbance in feeding or eating that results in severe malnutrition, significant weight loss or failure to gain weight, or growth compromise that impairs psychosocial functioning [1]. In ARFID, restrictive eating behaviors are associated with apparent lack of interest in eating, avoidance based on the sensory characteristics of food, or concerns about the aversive consequences of eating. Notably, eating behaviors in ARFID are not primarily motivated by weight or body image concerns [1]. ARFID commonly begins in early childhood and, if untreated, may persist into adulthood [14]. Point prevalence of full threshold ARFID based on self-reported symptoms was estimated at 0.8% for women and 0.9% for men [14]. Nakai et al. [22] found that ARFID accounted for 9.2% of all eating disorder (ED) hospital admissions (mixed adult and adolescent sample). Similar to anorexia nervosa (AN), patients with ARFID may experience medical complications and sometimes require hospitalization for nutritional rehabilitation [15, 22], but best practices for such interventions are yet to be defined. More research is needed to describe clinical presentations and illness course of adult ARFID to inform treatment guidelines.

Few studies examined ARFID symptomatology in adults. Zickgraf et al. [34] found that adult participants with ARFID symptoms endorsed high levels of quality of life impairment, internalizing symptoms and obsessive–compulsive symptoms. Another study observed adults with self-reported ARFID symptoms endorsed lower weight and shape-related ED symptomatology but similar levels of anxiety and depression as participants with other EDs,ARFID symptoms were unrelated to age and sex [14]. Both Thomas et al. [33] and Manwaring et al. [20] reported clinical levels of anxiety and depression in adults with ARFID. Fjeldstad et al. [8] compared adults with ARFID to those with AN and found lower self-reported anxiety symptoms and lower weight and shape-related ED symptomatology in adults with ARFID, but no differences in depression. Several other studies used mixed-age samples (adolescents and adults) to characterize ARFID. For example, Nakai et al. [22] found that individuals with ARFID scored lower than those with AN on drive for thinness, bulimic symptoms, body dissatisfaction, perfectionism, and interpersonal distrust. Becker et al. [3] also found that individuals with ARFID scored lower than those with AN on most measures of weight and shape-related psychopathology, except for restrictive eating. Although both anxiety and depression were lower in ARFID than AN, mean anxiety scores were within a clinical range [3]. Overall, it appears that individuals with ARFID experience similar impairment to those with other EDs, although more research in adults is needed.

Due to poor nutritional intake or dangerously low weight, patients with ARFID or AN may require hospitalization for normalization of eating behaviors and weight restoration. The majority of studies examining ARFID hospital course were in adolescent or mixed-age samples. Several studies of hospitalized patients with ARFID have found that adolescents and young adults with ARFID have similar body mass index (BMI) or percent target weight at hospital admission compared to that of patients with AN [8] (all adult sample); [18, 22, 30], but one study reported higher admission BMI in adolescents and young adults with ARFID [19]. The findings regarding length of stay are inconsistent, with one study reporting longer length of stay for a mixed age sample with ARFID versus AN [30] and another reporting no difference in length of stay [19]. Although one study observed a slower rate of weight gain in youth with ARFID compared with that of AN (1.36 kg vs. 1.92 kg/week respectively; [19]), another found no difference [30]. Adolescents and young adults with ARFID and AN showed comparable weight outcomes at hospital discharge (e.g., discharge BMI, % discharged for clinical improvement [18, 19]. Two studies have examined longer term outcomes post discharge, with one study finding that adolescents with ARFID had a higher rate of recovery at follow-up than did those with AN (77% ARFID and 43% AN recovered at 2-37 months post-discharge) [18]. The second study found similar remission rates at 1-year follow up (62% ARIFD and 46% AN remitted) although this difference did not reach statistical significance [32].

Overall, findings regarding hospital course and outcomes of patients with ARFID vs AN are inconsistent, which is likely due in part to very low sample sizes for the ARFID group in most of these studies (*N* range = 7–41). The sample sizes of AN and ARFID groups were also uneven, with the AN sample often being several times larger. Both these issues can introduce bias in the analyses. Further, all the studies of hospital course except for one [8] focused on children and adolescents or had a mixed age sample. More research on treatment course and outcomes in hospitalized adult patients with ARFID using larger samples is needed to inform treatment protocols and approaches to renourishment in patients hospitalized with underweight ARFID. This study compared inpatient treatment course of underweight adult patients with ARFID and sex- and age-matched patients with AN (total *N* = 138; 69 AN and 69 ARFID). To minimize sample bias, we performed case control matching based on sex and age to obtain samples similar in demographic characteristics. Because of inconsistent findings in the prior literature regarding treatment outcomes, no hypotheses were generated. We also compared self-reported symptomatology of patients with ARFID vs AN in a subset of the sample that had this data available (total *N* = 54; 27 AN and 27 ARFID). Despite the smaller sample size, these analyses were included due to the paucity of literature on adults with ARFID, although associated findings are preliminary and should be interpreted with caution. We hypothesized that patients with ARFID would report lower ED and depression symptoms than patients with AN. We did not make hypotheses regarding anxiety, desired weight, or personality traits because of conflicting findings or lack of prior research.

## Methods

### Participants

The sample consisted of 138 hospitalized underweight adult patients with ARFID or AN admitted to a specialized behavioral inpatient treatment program for eating disorders between 2003 and 2022. Patients with ARFID (*n* = 69) were matched to those with AN (*n* = 69) based on sex and age. A subsample of adults with ARFID (*n* = 27) who completed self-report questionnaires at admission were matched to 27 patients with AN based on sex and age (total sample with available self-report data *N* = 54). Sample demographics are presented in Table 1.

**Table 1:** Sample demographic and descriptive statistics characteristics

	Full Sample (*N* = 138)		Subsample^a^ (*n* = 54)	
	ARFID (*n* = 69)	AN (*n* = 69)	ARFID (*n* = 27)	AN (*n* = 27)
Age, M (*SD*)	35.86 (16.45)	35.86 (16.45)	32.96 (14.01)	32.96 (14.01)
BMI at admission, M (*SD*)	16.38 (1.85)	15.86 (1.91)	15.99 (1.55)	15.65 (1.90)
Length of stay (days), M (*SD*)	40.39 (26.42)	59.46 (31.11)	47.41 (22.75)	55.26 (27.35)
Sex, *n* (%)				
Female	61 (88.4%)	61 (88.4%)	23 (85.2%)	23 (85.2%)
Male	8 (11.6%)	8 (11.6%)	4 (14.8%)	4 (14.8%)
Ethnicity, *n* (%)				
Hispanic or latino	3 (4.3%)	0 (0%)	0 (0%)	1 (3.7%)
Not hispanic or latino	65 (94.2%)	67 (97.1%)	25 (92.6%)	25 (92.6%)
Chose not to disclose	1 (1.4%)	2 (2.9%)	2 (7.4%)	1 (3.7%)
Race, *n* (%)				
American Indian/Alaskan Native	0 (0%)	0 (0%)	0 (0%)	0 (0%)
Asian	1 (1.4%)	2 (2.9%)	0 (0%)	1 (3.7%)
Native Hawaiian or Pacific Islander	0 (0%)	0 (0%)	0 (0%)	0 (0%)
Black or African American	7 (10.1%)	0 (0%)	0 (0%)	2 (7.4%)
White or Caucasian	57 (82.6%)	66 (95.7%)	26 (96.3%)	22 (81.5%)
Multiracial	2 (2.9%)	0 (0%)	0 (0%)	1 (3.7%)
Other	1 (1.4%)	0 (0%)	0 (0%)	0 (0%)
Chose not to disclose	1 (1.4%)	1 (1.4%)	1 (3.7%)	1 (3.7%)

BMI, body mass index (kg/m^2^); %TW, percent target weight; AN, anorexia nervosa; ARFID, avoidant/restrictive food intake disorder. ^a^A subset of patients from the full sample consented to complete additional self-report questionnaires at admission

### Procedures

Clinical and demographic data were collected as part of routine clinical care. A retrospective chart review approved by the Institutional Review Board of the Johns Hopkins University School of Medicine allowed abstraction of de-identified demographic and clinical data from the electronic medical record. This data included admission height and weight, pounds from target weight at admission, weight at discharge, length of stay (in days), achievement of target weight (yes or no), and reason for discharge (for clinical improvement vs not for clinical improvement [e.g., against medical advice, insurance, etc.]). Additional self-reported questionnaire data were collected from a subset of these patients who consented to participate in a longitudinal outcomes research study. Questionnaire data were collected using paper and pencil within the first week of admission. ED diagnoses were determined via the Structured Clinical Interview for the Diagnostic and Statistical Manual of Mental Disorders, 5th Edition (DSM-5; [7]). Diagnostic interviews were administered by postdoctoral fellows or trained research assistants supervised by a licensed clinical psychologist. Prior to the existence of DSM-5 and the introduction of the ARFID diagnosis, this study would have classified ARFID cases as Eating Disorder Not Otherwise Specified. After the release of DSM-5, all diagnoses were reviewed by a team of psychiatrists and clinical psychologists to ensure diagnoses consistently reflected the revised DSM-5 criteria. The SCID was administered by only one rater, and therefore inter-rater reliability is not available.

### Treatment protocol

The inpatient eating disorders program employs a structured meal-based behavioral treatment protocol delivered within a multidisciplinary integrated, inpatient program. Primary treatment goals include rapid weight restoration for underweight patients and normalization of eating behavior. The nutritional protocol is 100% meal-based and nasogastric feeds are never employed. Patients consume three supervised meals a day in a group setting. Calories are advanced from 1200 to 2000 kcals/day (depending on admission BMI) to 3500–4000 kcals/day for individuals on weight gain protocol [11]. Calories above 2500 are administered via snacks and liquid supplements. With rare exceptions (e.g., patient was vegetarian at least three years prior to developing an ED, religious exceptions, or documented food allergy), food preferences or dislikes are not accommodated, as the goal is to help patients diversify their food intake. Patients may begin selecting menu items contingent upon completing 100% of meals served once at a calorie intake level of 3500 cal/day typically by day 10–12. Menu selections are reviewed by staff for compliance with the exchange plan. The nutritional rehabilitation protocol was identical for patients with ARFID and AN.

### Measures

#### Weight and BMI

Height and weight measured at admission and discharge were used to compute admission and discharge BMI. Individualized target weights set for each patient were a four-pound range (1.8 kg) based on the patient’s age, sex, and height centered on a BMI of 20.5 kg/m^2^ for patients over age 25. For patients aged 18–24, target weight was determined using growth charts when available. For patients whose baseline BMI was above the 50th percentile target BMI was set at the 50th BMI percentile. For those whose baseline BMI curve was below the 50th percentile but above the 25th, target BMI was calculated to fall on their premorbid BMI curve and for patients whose baseline BMI trajectory was below the 25th percentile, target BMI was set to the 25th BMI percentile. When growth charts were unavailable, target weight was set using the formula for adults over the age of 25 and adjusted by subtracting one pound (0.45 kg) per year of age below 25. Desired weight was assessed as a part of self-report questionnaires with one item (“How much would you like to weigh?”).

#### Eating disorder inventory-2 (EDI-2)

The EDI-2 is a 64-item self-report measure of cognitive and behavioral characteristics of weight and shape-related eating disorders [9]. Three subscales were used in the current study: Drive for Thinness, Bulimia, and Body Dissatisfaction. The EDI-2 has demonstrated good validity and reliability [9, 32] in patients with AN and BN, but no data are available on psychometric properties of the EDI-2 in patients with ARFID. Internal consistency for Cronbach’s alphas for Drive for Thinness, Bulimia, and Body Dissatisfaction were excellent in this study (*α* = 0.94, *α* = 0.83, and *α* = 0.91).

#### Eating disorder recovery self-efficacy questionnaire (EDSRQ)

The EDSRQ is a 23-item self-report measure of self-efficacy to refrain from acting on eating disordered behaviors and attitudes [21]. The Normative Eating Self-Efficacy subscale assesses confidence to eat without engaging in disordered eating behaviors (e.g., restricting, binge eating) and the Body Image Self-Efficacy subscale assesses confidence to maintain a realistic body image not dominated by pursuit of thinness. The EDRSQ has demonstrated good validity and reliability among individuals with weight and shape-related eating disorders [21, 24], but no psychometric data is available in ARFID samples. Internal consistencies for Normative Eating Self-efficacy and Body Image Self-efficacy were excellent in this study (*α* = 0.97, *α* = 0.93).

#### State and trait anxiety inventory (STAI)

The STAI is a 40-item self-report measure of anxiety as experienced in the moment (state subscale; STAI-S) and as a stable personality trait (trait subscale; STAI-T; [27]). The STAI has demonstrated good reliability and validity [12, 23]. Internal consistency for state and trait anxiety were excellent in the current study (*α* = 0.97, *α* = 0.94).

#### Beck depression inventory-II (BDI-II)

The BDI-II [2] is a 21-item self-report measure of depressive symptomatology. The BDI-II has strong psychometric properties, including internal consistency and factor validity [2, 28]. Internal consistency for the BDI-II in this study was excellent (*α* = 0.91).

#### NEO five-factor inventory (NEO-FFI)

The NEO-FFI [5] is a widely-used 60-item self-report questionnaire used to provide a concise measure of the big five personality factors including neuroticism, extraversion, openness to experience, agreeableness, and conscientiousness. The NEO-FFI has demonstrated adequate psychometric properties among individuals with weight and shape-related eating disorders [31]. Internal consistencies in the current study were excellent for neuroticism (*α* = 0.91), good for extraversion (*α* = 0.80) and conscientiousness (*α* = 0.87) and acceptable for openness to experience (*α* = 0.76) and agreeableness (*α* = 0.61).

### Data analysis

#### Power analysis

According to G*Power [6], a sample size of n = 64 in each group is required to detect a moderate effect size and a sample size of n = 26 is required to detect a large effect using an independent t-test analysis (power = 0.80, α = 0.05).

#### Treatment course

All analyses were conducted in SPSS v 28. The total sample (*N* = 138) was used for treatment course and outcomes analyses. Independent t-tests were conducted to compare patients with ARFID and AN on the following variables: Admission BMI, pounds from target weight at admission, length of stay, rate of weight gain, and discharge BMI. Pearson chi-square tests examined whether groups differed in whether they achieved target weight at discharge and reasons for discharge (clinical improvement vs not clinical improvement). Clinical improvement indicated that the patient had made sufficient progress in treatment and was ready to step down to a lower level of care for continued treatment and weight restoration. Not for clinical improvement indicated that the patient was discharged for other reasons including financial concerns, patient/family request (against medical advice), administrative discharge for non-compliance, transfer to medical or other psychiatric unit, or elopement.

#### Self-reported symptomatology

The subsample of patients who completed self-report questionnaires (*N* = 54) was used for these analyses. Independent t-tests were conducted to compare patients with ARFID and AN on the following admission variables: weight and shape-related ED symptoms, normative eating and body image self-efficacy, desired weight, anxiety, depression, and personality traits.

## Results

### Treatment course

Although patients with ARFID did not significantly differ from patients with AN on admission BMI, they were closer to their target weight at admission (Table 2). We conducted a post-hoc test comparing target weight of patients with ARFID and AN and found that for those under the age of 25, the ARFID group had lower target weights compared to AN (*t*(136) = 3.30, *p* = 0.002). No group differences in target weight were found for adults 25 and older with AN and ARFID (*p* = 0.636). Groups did not significantly differ in length of stay. However, patients with ARFID gained weight at a significantly slower rate, had lower discharge BMI, and were less likely to reach target weight by discharge than patients with AN (χ2[1, 138] = 9.44, *p* = 0.002). Sixty percent of patients with AN and only 33% of patients with ARFID reached target weight before discharge. There were no differences between groups on reason for discharge (clinical improvement vs not clinical improvement; χ^2^[1, 138] = 0.26, *p* = 0.607). Fifty eight percent of patients with AN and 54% of patients with ARFID were discharged for clinical improvement. Out of 29 patients with AN discharged not for clinical improvement, 14 discharges were rated as patient or family-initiated, five were for financial reasons, five for non-compliance, three were transfers to other medical or psychiatric units, and two patients eloped. Out of 31 patients with ARFID discharged not for clinical improvement, 18 discharges were rated as patient or family-initiated, two were for financial reasons, five were for non-compliance, and six were transfers to other medical or psychiatric units.

**Table 2:** Comparison of patients with ARFID vs AN

Treatment course	ARFID (*n* = 69)	AN (*n* = 69)		
	*M (SD)*	*M (SD)*	*t*	*p*
Admission BMI	16.38 (1.85)	15.86 (1.91)	− 1.63	.106
Percent target weight (Admission)	79.39(8.68)	77.19(9.75)	− 1.36	1.74
Pounds from target weight	− 23.41 (11.42)	− 28.14 (12.54)	− 2.31	.022
Rate of weight gain (lbs per week)	3.04 (2.02)	4.09 (1.35)	3.59	< .001
Length of stay (days)	31.43 (19.55)	34.51 (21.28)	.88	.379
Discharge BMI	19.14 (2.02)	20.01 (1.54)	2.79	.006

Admission characteristics	ARFID (*n* = 27)	AN (*n* = 27)		
	*M (SD)*	*M (SD)*	*t*	*P*
EDI-2 Drive for thinness	11.62 (3.48)	31.50 (10.45)	10.99	< .001
EDI-2 Bulimia	9.82 (3.74)	15.64 (8.46)	3.84	< .001
EDI-2 Body dissatisfaction	23.96 (6.52)	39.69 (1.79)	6.14	< .001
EDRSQ—Normative eating self-efficacy	4.05 (.86)	2.06 (1.10)	− 7.76	< .001
EDRSQ—Body image self-efficacy	3.68 (.74)	1.91 (.91)	− 7.82	< .001
Desired BMI	20.46 (2.20)	17.41 (2.59)	− 2.70	.010
STAI-state anxiety	43.36 (14.47)	61.40 (11.08)	2.58	.017
STAI-trait anxiety	44.47 (12.61)	56.80 (13.36)	1.92	.068
BDI-Depression	20.69 (10.87)	30.11 (12.93)	2.84	.006
NEO—Neuroticism	23.85 (7.96)	33.26 (11.01)	3.58	< .001
NEO—Extraversion	26.85 (8.18)	23.46 (6.91)	− 1.63	.110
NEO—Openness	31.15 (5.90)	28.03 (7.03)	− 1.75	.087
NEO—Agreeableness	33.37 (5.59)	31.42 (6.24)	− 1.19	.237
NEO—Conscientiousness	32.25 (8.49)	31.69 (7.63)	− .26	.800

ARFID, avoidant/restrictive food intake disorder; AN, anorexia nervosa; BMI, body mass index (kg/m^2^); EDI, Eating Disorder Inventory; EDSRQ, Eating Disorder Recovery Self-Efficacy Questionnaire; STAI, State-Trait Anxiety Inventory; BDI, Beck Depression Inventory

### Self-reported symptomatology

Patients with ARFID scored lower on ED symptoms, state anxiety, depression, and neuroticism and higher on Normative Eating and Body Image Self-efficacy (Table 2) compared to those with AN. There were no differences between groups on trait anxiety or other personality variables (i.e., openness to experience, etc.). Patients with ARFID endorsed a higher desired BMI than patients with AN.

## Discussion

Little is known about the presentation and treatment of ARFID in adults. The current study compared treatment course and self-reported symptomatology in underweight adults with ARFID admitted to an inpatient ED behavioral unit to age and sex-matched adults with AN. Adults with ARFID endorsed less psychopathology, including ED symptoms, state anxiety, depression, and neuroticism than patients with AN. Despite being closer to target weight, adults with ARFID gained weight at a slower rate, were discharged at lower BMI, and were less likely to reach target weight by discharge than adults with AN. While these results may suggest less favorable inpatient outcomes for adults with ARFID, patients in both groups were equally likely to be discharged for clinical improvement. Follow-up data is needed to evaluate short- and long-term outcomes of inpatient treatment for adults with ARFID.

### Treatment course

Consistent with prior literature, we found that adults with ARFID had similar BMI at admission to those with AN (e.g., [8, 18, 22]). Adults with ARFID had less weight to gain to reach their target weight, which was explained by lower average goal weight ranges for ARFID compared to AN in those aged 18–25 years. Growth charts would have been incorporated into setting target weight for this age group if available. It is possible that a lower target weight was set for a subset of patients with ARFID who had a lifetime history of low weight. However, growth charts were not available to test this hypothesis. Steinberg et al. [29] also found that an individualized approach to setting target weight (using growth charts) resulted in lower target weight in children and adolescents with ARFID compared to other ED diagnoses. Further investigation on this issue is warranted. Like Makhzoumi et al. [19], we found no differences in length of stay between adults with ARFID and AN, but patients with ARFID gained weight at a slower rate than those with AN. These similarities are not surprising considering both studies used data from patients admitted to the same treatment program (samples were partially overlapping).

While prior literature suggested similar discharge BMI for samples of adolescents and young adults with ARFID and AN [18, 19], we found that adults with ARFID had lower BMI at discharge and were less likely to achieve target weight by the end of their hospital stay than patients with AN. Only one third of ARFID patients reached target weight by discharge, compared with 60% of patients with AN. However, similar proportions were discharged for clinical improvement in both groups. Because patients with ARFID do not fear gaining weight, a treatment team may be willing to discharge them prior to reaching target weight, expecting the patients will continue to gain weight in less restrictive levels of care (i.e., outpatient). Future studies should examine whether personalizing treatment length based on early response in hospitalized patients with ARFID is predictive of outcome.

The slower rate of weight gain observed in the ARFID group may reflect that those with ARFID had more difficulty with meal completion compared to patients with AN, which may be due to the fact that the nutritional rehabilitation protocol was designed for patients with AN. Similar to other ED treatment programs [13] and due to a lack of existing ARFID treatment guidelines, this treatment center uses an AN treatment protocol for patients with ARFID. Programs that adapted an AN treatment protocol to treat ARFID reported that ARFID patients required a more gradual exposure to non-preferred foods than those with AN [25],these modifications were not part of the current treatment protocol. Additionally, nasogastric (NG) feeds are commonly used for nutritional rehabilitation in ARFID, with one study reporting higher use of NG tubes in patents with ARFID compared to AN [30]. However, our program does not employ NG feeds for any patients. Although this study does not have the data on ARFID subtype, prior literature suggests that hospitalized patients with ARFID are more likely to suffer from fear of aversive consequences (e.g., choking; [16]. Existing protocols may need to be adapted to meet the unique needs of individuals with ARFID, which would depend on the disorder subtype (e.g., sensory sensitivities, lack of interest in food, or fear of aversive consequences). For example, adding an exposure intervention to specifically target patients’ fears may be beneficial. Additionally, individualized treatment protocols may be needed for patients with ARFID to address life-long dietary restriction, improve efficacy, and prevent relapse. Nutritional rehabilitation for patients with ARFID should be further studied to inform development of specialized treatment protocols for this population.

### Self-reported symptomatology

As expected, adults with ARFID endorsed lower levels of drive for thinness, bulimia, and body dissatisfaction and higher levels of normative eating and body image self-efficacy. The normative eating and body image self-efficacy subscales of the EDRSQ assess difficulties with eating and body image related to fear of fatness specifically [21], which would explain relatively lower scores in the ARFID population. Adults with ARFID reported having higher desired BMI than patients with AN. Notable, unlike in the AN group, desired BMI was within the normal range, consistent with absence of fear of fatness in ARFID. Consistent with prior research [4, 8], adults with ARFID had lower state anxiety and depression than those with AN, but groups did not differ in trait anxiety. It is possible that patients with AN have higher state anxiety at admission due to fear of gaining weight during their hospitalization, which would not be present in patients with ARFID.

Adults with ARFID and AN did not differ on openness to experience, extroversion, conscientiousness, or agreeableness, although we only had enough power to detect a large effect size. However, adults with ARFID reported significantly lower neuroticism. Neuroticism is a disposition to experience negative affect and is the core dimension of internalizing psychopathology, which includes anxiety, depressive, and weight and shape-related eating disorders [10]. Lower neuroticism in adults with ARFID than in AN may highlight that these disorders are associated with different dimensions of psychopathology. AN is part of an internalizing disorders spectrum characterized by negative emotions [17]. Although ARFID has not been classified within dimensional models of psychopathology, some consider it to be similar to neurodevelopmental disorders, such as autism spectrum disorder and attention deficit hyperactivity disorder, with which ARFID is highly comorbid [26]. It is possible that different ARFID subtypes (i.e., sensory difficulties, fear of aversive consequences, lack of interest in food) may have different comorbidities and associations with psychopathology symptoms, which future research should investigate.

## Limitations and conclusions

Several limitations should be noted. First, our sample size for the self-reported symptomatology analyses was small, and these results should be viewed as preliminary. Second, we did not include measures of ARFID-specific psychopathology in this study. Future studies should include such assessments in addition to more traditional eating disorder weight and shape-related measures. Third, all analyses are correlational, and no causal relationships can be implied. Fourth, the samples were primarily Non-Hispanic Whites and the results may not generalize to minority populations. Fifth, as these were treatment-seeking underweight adults with ARFID and AN results may not generalize to other ARFID populations. Sixth, we do not have data on ARFID subtypes (i.e., sensory difficulties, fear of aversive consequences, lack of interest in food), and findings may be affected by one subtype being more prevalent than the others. Sixth, we do not have follow-up data and are not able to draw conclusions regarding treatment outcome beyond discharge from inpatient treatment. Seventh, our sample is primarily female and the results may not generalize to male patients with ARFID. Finally, since length of illness is associated with ED severity, it may be important to match participants with ARFID and AN on this variable in addition to age and sex. Unfortunately, data on length of illness was not available for the current study.

This study adds to limited literature on presentations of ARFID in adults, specifically focusing on an underweight hospitalized population. Despite being closer to target weight at admission and reporting less comorbid psychopathology, adults with ARFID had less favorable weight-related outcomes during inpatient hospitalization, but long-term outcomes are unclear. More research is needed to better understand adult ARFID presentations to improve existing interventions.

## Data Availability

The data that support the findings of this study are available on request from the corresponding author. The data are not publicly available due to privacy or ethical restrictions.
